# Changes of empathy in medical college and medical school students: 1-year follow up study

**DOI:** 10.1186/1472-6920-12-122

**Published:** 2012-12-17

**Authors:** Minha Hong, Won Hye Lee, Jae Hyun Park, Tai Young Yoon, Duk Soo Moon, Sang Min Lee, Geon Ho Bahn

**Affiliations:** 1 Department of Neuropsychiatry, School of Medicine, Kyung Hee University, 1 Hoegi-Dong, Dong Dae Moon-Gu, 130-702, Seoul, South Korea; 2 Department of Medicine, Graduate School, Kyung Hee University, 1 Hoegi-Dong, Dong Dae Moon-Gu, 130-702, Seoul, South Korea; 3 Department of Medical Education, School of Medicine, Kyung Hee University, 1 Hoegi-Dong, Dong Dae Moon-Gu, 130-702, Seoul, South Korea; 4 Department of Preventive Medicine, School of Medicine, Kyung Hee University, 1 Hoegi-Dong, Dong Dae Moon-Gu, 130-702, Seoul, South Korea

**Keywords:** Empathy, Medical college, Medical school, Medical education

## Abstract

**Background:**

This study aims to determine the correlation between medical education systems, medical college (MC) and medical school (MS), and empathy by investigating the changes in empathy among students with each additional year of medical education.

**Methods:**

The subjects were MC and MS students who had participated in the same study the previous year. All participants completed the same self-report instruments: a questionnaire on sociodemographic characteristics, and the Korean edition of the Student Version of the Jefferson Scale of Empathy (JSE-S-K), Among 334 students, the final analysis was conducted on the data provided by 113 MC and 120 MS students, excluding 101 with incomplete data.

**Results:**

The age and sex did not affect the changes in empathy. Though the JSE-S-K score of MS was significantly higher than that of MC in initial investigation, this study found no difference of empathy between MC and MS.

**Conclusion:**

Empathy increased significantly after one year of medical education. The difference between two education systems, MC and MS, did not affect the changes in empathy.

## Background

It is important to determine the quality as well as the technical aspects of medical services. Empathy plays a key role in positive doctor-patient relationship and patient satisfaction, and contributes to optimal clinical outcomes [1,2].

In medical education, empathy is considered to be an essential professional characteristic for clinicians [3]. One of the goals in a curriculum for medical education includes improvement of empathy [4]. There has been a number of studies reporting on changes in empathy and empathy-related factors after medical training experience [5-10]. Many of them [6-8]^.^ reported that a student’s empathy declined during medical school education and that one’s attributes related to empathy, such as humanitarianism, enthusiasm and idealism, also decreased. According to a study on empathy of medical school students in the U.S. conducted with the Jefferson Scale of Physician Empathy-Student Version (JSPE-S) [8], the empathy scores declined significantly during their 3rd year, which was their first full year of clinical experience. A study at Boston University School of Medicine [6] also found that empathy scores of the U.S. medical school students on the JSPE-S went down in their clinical years: the empathy scores increased during the year after school entrance, decreased slightly in the second year and decreased significantly in the third year (first clinical year). A further study conducted at Jefferson Medical College reported a similar finding; while empathy scores did not alter significantly during the first two years (preclinical years), they decreased during the 3rd year (first clinical year) and remained low until graduation [7].

However, a recent study on empathy performed with medical school students in Japan showed that the empathy increased between their first year and the end of training year [9]. A study on empathy of medical school students conducted at a Korean medical school also revealed that later years of training were associated with significantly better empathy [10]. One explanation for these different findings is that empathy increased as a result of differences in medical education system, but there has been insufficient number of studies to be confident that such a causal relationship exists.

The medical education system in Korea has gone through a period of transition. Most Korean medical colleges take a 6-year curriculum with 2-year of premedical course & 4-year medical course; the newly adopted medical schools take a 4-year curriculum, much similar to that of the US. There is no regulation on undergraduate major of MS students before entry into medicine. We call an existing 6-year medical curriculum as medical college (MC) and another new medical education system with a 4-year medical course as medical school (MS).

In 2-year of premedical course, medical college (MC) students take classes of basic sciences such as biology, cell biology, and comparative anatomy, and of introductions to psychology, oriental medicine, dental medicine, medicine, medical statistics, medical informatics, and behavioral science. The curriculum of 4-year of medical courses are almost the same in two systems, except that 1 or 2 classes about medical research and English are taken in medical school (MS).

In the initial investigation, the third-year students of both systems had significantly higher scores on the JSE-S-K than the first-year students and MS students had significantly higher scores on the JSE-S-K than MC students. The present study represented a follow-up to an initial investigation of comparing two medical education systems [10] and aimed to explore further the relationship between the medical education systems, of medical colleges (MC) and medical schools (MS), and empathy, by investigating the changes in the same groups of students after each year of medical education to determine whether the difference in empathy originally found between the two medical education systems was maintained one year later or not.

## Methods

### Subjects and data collection

The subjects were MC and MS students in the same medical course in the second, third and fourth year at Kyung Hee University in 2008, who had participated in the same questionnaire survey one year previously, in 2007 [10].

As was the case in the first questionnaire survey in 2007 [10], the students filled in the questionnaires just after the final examination of the second semester, in 2008. A total of 200 MC and 152 MS students were recruited for the first survey in 2007, but 57 students with incomplete answers were excluded, leaving the data of 155 MC and 137 MS students for the final analysis. The participants of this follow-up survey were 334 students, comprised of 187 MC and 147 MS students. After excluding those who provided incomplete data in the first survey (n = 57) and those in the follow-up survey who did not return their questionnaires (n = 31) or supplied incomplete data (n = 13), the final analysis for the current study was conducted on 113 MC and 120 MS students. The average age of MS students was significantly higher than that of MC students (27.0 ± 2.6 vs. 23.8 ± 1.7, p < 0.05), and the percentage of females of the participants was higher in MS students (62.5% vs 24.8%, p < 0.05). This study was approved by Institutional Review Board of Kyung Hee University Hospital.

### Measurement tools

#### Korean edition of the Student Version of the Jefferson Scale of Empathy (JSE-S-K) [11]

The JSE is a scale developed by Hojat et al. [12] to assess empathy of medical school students (JSE-S) and physicians and health professionals (JSE-HP). The JSE-S-K is a self-report rating scale consisting of 20 items covering 18 areas of emotion and two cognitive areas. Each question is answered on a seven-point Likert scale, and the maximum score is 140 with higher scores indicating higher empathy. The internal consistency (Cronbach’s alpha) of the JSE-S-K was reported to be 0.82 (p < 0.05), 0.85 (p < 0.05) and 0.73 (p < 0.05) for the total scale, and the emotion and cognition items, respectively [11].

## Data analysis

Chi-square and Student’s t tests were conducted to examine the differences in socio-demographic variables between MC and MS students. A mixed effect ANOVA was performed to investigate changes in JSE-S-K scores over one year by medical education system and year of study. A p-value of <0.05 was considered to indicate statistical significance.

## Results

### Sociodemographic characteristics and changes in empathy

Male and female students did not differ (p = 0.95) on changes in JSE-S-K scores between the first and second survey (Table 1). The correlation of the age of students with change in empathy was not significant (r = −0.045, p = 0.494).

**Table 1:** Comparison of changes of JSE-S-K scores by sex

**Sex**	Past^*^Mean(**SD)**	**Present**^**†**^**Mean**(**SD)**	**Δ Mean**(**SD)**	**t**	**p**
Male	107.33(10.59)	109.24(11.74)	1.91(11.40)		
Female	109.15(11.08)	111.14(10.55)	2.00(11.65)		
Total	108.14(10.83)	110.09(11.25)	1.95(11.50)		

JSE-S-K: Jefferson Scale of Empathy, S-version, Korean edition, SD: Standard deviation.

^*^past means survey on 2007.

^†^present means survey on 2008.

### Changes in empathy by year of training and medical education system

A mixed ANOVA was performed to examine changes in empathy scores over one year between grades and medical education systems. The main effect of time (F = 6.81, p = .01) was statistically significant, but none of the interaction effects were significant among within-subject variables. There were no significant differences among between-subject variables. These results shows that empathy scores significantly increased over the one-year period between 2007 and 2008 surveys regardless of year of training or medical education system (Table 2).

**Table 2:** Comparison of JSE-S-K scores between MC and MS

**Grade**	Past^*^Mean(**SD)**	**Present**^**†**^**Mean**(**SD)**	**Δ Mean**(**SD)**	**t**	**p**	***Cohen’s d***
**MC (Past → Present)**						
1st **→** 2nd (n = 31)	104.57(10.40)	108.43(13.29)	3.86(12.01)	−1.76	0.09	*0.32*
2nd **→** 3rd (n = 38)	107.05(11.27)	109.31(9.96)	2.26(10.97)	−1.14	0.26	*0.18*
3rd **→** 4th (n = 44)	108.52(9.79)	111.34(10.47)	2.81(9.43)	−1.98	0.05	*0.30*
*MC total (n = 113)*	*106.96(10.50)*	*108.88(11.11)*	*2.92(11.08)*	*−2.78*	*0.01*	*0.26*
**MS (Past → Present)**						
1st **→** 2nd (n = 43)	107.79(12.00)	109.58(10.93)	1.79(10.97)	−1.07	0.29	*0.16*
2nd **→** 3rd (n = 40)	109.05(11.97)	110.17(13.06)	1.12(14.51)	−0.49	0.63	*0.08*
3rd **→** 4th (n = 37)	111.11(8.61)	111.24(10.27)	0.13(9.74)	−0.08	0.93	*0.01*
*MS total (n = 120)*	*109.23(11.06)*	*110.29(11.43)*	*1.06(11.86)*	*−0.98*	*0.33*	*0.09*
**Total (n = 233)**	108.14(10.83)	110.09(11.25)	1.95(11.50)	−2.59	0.01	*0.17*

JSE-S-K: Jefferson Scale of Empathy, S-version, Korean edition, SD: Standard deviation. MC: Medical College Students. MS: Medical School Students.

^*^past means survey on 2007.

^†^present means survey on 2008.

## Discussion

The present study found that changes in empathy were unrelated to gender. Similar findings were reported in the study by Lee et al. [10] on Korean medical school students and one by Hong et al. on Korean psychiatric residents [13]. The conflicting results compared to previous Japanese, American, and Italian studies [9,14,15] are considered to be come from cultural differences. Another effect of cultural differences is the mean scores of JSE-S. In this and previous studies of Korea [10], the mean JSE-S scores were 108.14 and 110.09, which were lower than that of the U.S., range of 109 to 123 [5,7,8]. It is assumed that the feeling and expression of empathy are culturally varied, and therefore essential further global studies to demonstrate.

There has been inadequate amount of attention paid to the literature on empathy development in medical education, leading to a lack of knowledge on how medical education may modulate empathy [16]. Further research is needed to examine this hypothesis.

This follow-up study, which was conducted one year after the initial study by Lee et al. [10] found that no significant score differences in JSE-S-K scores between the two education systems. This important finding is that empathy improved significantly after one year of medical education, regardless of the year of study and the medical education system.

After a thorough examination, it was suggested that a class entitled ‘Physicians in society’, taken for 3 years by both MC and MS students, might have a positive effect on empathy development. This class is worth one credit and is a required subject for one hour per week. The class deals with medical ethics, doctor-patient relationships, and communication; the course contents are thought to improve the students’ attitudes towards patients, their understanding of ethics, and their capacity for empathy. Although their course content was not specifically related to medical practice, Fernández-Olano et al. [17] showed that a 25-hour workshop program involving training in general communication and empathy skills increased the JSPE scores of medical students and medical residents by 5.31 points. In review of 11 relevant studies, Colliver et al. [13] showed that the evidence does not warrant the strong, disturbing conclusion that empathy declines during medical education. Like the studies by Lee et al. [10] and Colliver et al. [13], this study found no negative effect of clinical experiences on empathy. Hong et al. [18] showed empathy in Korean psychiatric residents was higher in senior residents. Cultural or educational differences between the East and the West could be assumed to be the reason for these conflicting results, and the positive factors for improving empathy needed to be determined. Furthermore, the exact mechanism how the class 'Physicians in society', affects empathy should be determined in future studies comparing groups taking the class or not.

In addition, the finding that every student group did show an absolute change but only individual year (MC students 3rd to 4th ) had a statistically significant change in JSE-S-K scores may be interpreted in point of the effect size, Cohen’s d value (Table 2) [19]. The MC student groups showed small effect sizes while the MS student groups did not reach meaningful level. In other words, the MC groups improved more than MS groups during a year of medical education while MS groups showed higher JSE-S-K scores than MC groups at initial investigation. Overall, a maturation effect resulting from age gap between two systems cannot be ruled out and should also be examined in further studies.

One of limitations of this study was that it was conducted at a single medical school and a single medical college rather than on a representative sample of national MC and MS students. In future studies, a more representative sample of students from regional or national schools of medicine is necessary. A second limitation is indirect measure of empathy by using JSE-S-K assessing beliefs or attitudes about the importance of empathy and emotion in medical practice. The specific relation of the JSE-S-K in real medical practice may be examined by complementary tools such as supervisor ratings or peer reviews in further research. The third limitation is that the participants of 2007 and 2008 studies were not exactly the same group as they completed the questionnaire anonymously. However, Tsimtsiou et al. [20] showed that no significant demographic differences were noted as the sample source was the same.

In spite of these deficiencies, this follow-up study improves upon the cross-sectional limitations of the initial study [10] by providing an evaluation of changes in empathy over a one-year period.

## Conclusion

The main finding of this study was that empathy in medical students increased significantly after one year of medical education. The differences between gender, age and in particular, medical education systems did not affect the changes in empathy.

## Competing interests

The authors declare that they have no competing interests.

## Authors’ contributions

All seven authors have made contributions to the study design, acquisition and interpretation of data. HMH and BGH drafted the manuscript, participated in design of the study and carried out the analysis. MDS and LSM assisted in writing the manuscript. LWH was responsible for the coordination of data and performed the statistical analysis. YTY and PJY made substantial contributions to the analysis and interpretations. All authors have been involved in revising the manuscript critically for important intellectual content and have read and approved the final manuscript.

## Pre-publication history

The pre-publication history for this paper can be accessed here:

http://www.biomedcentral.com/1472-6920/12/122/prepub
